# Yellow fever in Asia–a risk analysis

**DOI:** 10.1093/jtm/taab015

**Published:** 2021-01-28

**Authors:** Bethan Cracknell Daniels, Katy Gaythorpe, Natsuko Imai, Ilaria Dorigatti

**Affiliations:** MRC Centre for Global Infectious Disease Analysis; and the Abdul Latif Jameel Institute for Disease and Emergency Analytics (J-IDEA), School of Public Health, Imperial College London; MRC Centre for Global Infectious Disease Analysis; and the Abdul Latif Jameel Institute for Disease and Emergency Analytics (J-IDEA), School of Public Health, Imperial College London; MRC Centre for Global Infectious Disease Analysis; and the Abdul Latif Jameel Institute for Disease and Emergency Analytics (J-IDEA), School of Public Health, Imperial College London; MRC Centre for Global Infectious Disease Analysis; and the Abdul Latif Jameel Institute for Disease and Emergency Analytics (J-IDEA), School of Public Health, Imperial College London

**Keywords:** Arbovirus, Travel, Flavivirus, Aedes, Modelling, Surveillance, Outbreak

## Abstract

**Background:**

There is concern about the risk of yellow fever (YF) establishment in Asia, owing to rising numbers of urban outbreaks in endemic countries and globalisation. Following an outbreak in Angola in 2016, YF cases were introduced into China. Prior to this, YF had never been recorded in Asia, despite climatic suitability and the presence of mosquitoes. An outbreak in Asia could result in widespread fatalities and huge economic impact. Therefore, quantifying the potential risk of YF outbreaks in Asia is a public health priority.

**Methods:**

Using international flight data and YF incidence estimates from 2016, we quantified the risk of YF introduction via air travel into Asia. In locations with evidence of a competent mosquito population, the potential for autochthonous YF transmission was estimated using a temperature-dependent model of the reproduction number and a branching process model assuming a negative binomial distribution.

**Results:**

In total, 25 cities across Asia were estimated to be at risk of receiving at least one YF viraemic traveller during 2016. At their average temperatures, we estimated the probability of autochthonous transmission to be <50% in all cities, which was primarily due to the limited number of estimated introductions that year.

**Conclusion:**

Despite the rise in air travel, we found low support for travel patterns between YF endemic countries and Asia resulting in autochthonous transmission during 2016. This supports the historic absence of YF in Asia and suggests it could be due to a limited number of introductions in previous years. Future increases in travel volumes or YF incidence can increase the number of introductions and the risk of autochthonous transmission. Given the high proportion of asymptomatic or mild infections and the challenges of YF surveillance, our model can be used to estimate the introduction and outbreak risk and can provide useful information to surveillance systems.

## Introduction

Yellow fever (YF) is a zoonotic disease affecting individuals living in and travelling to the tropical and subtropical regions of Africa and South America.^1^ It is an arbovirus of the *Flavivirus* genus, maintained through up to three different transmission cycles.^2^ Of concern is the urban cycle, which occurs through sustained human-to-human transmission by the vector *Aedes aegypti* and can result in explosive outbreaks.^3^ These outbreaks can lead to large loss of life due to the severity of YF, with a case fatality rate of 67% reported amongst hospitalised cases.^4^ Diagnosis of YF is difficult as the clinical spectrum ranges from asymptomatic infection to fatal disease.^1^ Although an efficacious vaccine exists, there are global shortages due to difficulties in scaling-up vaccine production and the increasing number of YF outbreaks requiring response vaccination, which limits the extent to which routine immunisation can be conducted in several parts of Africa and South America.^5^^,^^6^

Critically, a 2016 outbreak in Angola resulted in the first recorded introduction of YF into Asia, after infected workers returned home to China.^7^ There was no onward transmission in China; however, >2 billion individuals in Asia live in areas infested with *A. aegypti* and *Aedes albopictus,* another competent and highly adaptable vector.^8–12^ Additionally, large parts of Asia are hyperendemic or have recorded outbreaks of dengue, chikungunya and Zika, which are transmitted by *Aedes* mosquitoes, suggesting conditions are also supportive for YF virus transmission. Whilst the circulation of dengue virus, chikungunya virus and Zika virus in Asia has been recorded since the mid-20th century, all three have become significant public health concerns in the last 50 years. Dengue incidence has increased 30-fold, with over half the global burden in South and South East Asia alone.^13^^,^^14^ Chikungunya has expanded globally since 2005, with widespread outbreaks across Asia.^15^ Finally, many Asian countries recorded Zika virus introduction and autochthonous transmission following the 2016 epidemic in the Americas.^16^^,^^17^

Factors driving the global spread of these arboviruses include urbanisation, climate change and unsustainable vector control, all of which facilitate the expansion of *Aedes* mosquito habitats.^18^ Globalisation also means that distance is no longer a limiting factor in the spread of disease and should an individual travel whilst infectious or incubating a virus there is a risk of them seeding an outbreak in a novel location.^19^^,^^20^ Critically, these demographic, entomological and epidemiological factors are also increasing the potential risk of YF transmission, suggesting YF could be next to spread globally.^21^ As vaccination coverage is near non-existent outside of endemic countries, an outbreak in Asia could require hundreds of millions of doses to mitigate an epidemic.^22^ In recognition of the major public health threat that the international spread of YF poses, many countries require arriving travellers to have a certificate of vaccination.^6^ However, the introduction of cases into China from Angola, both countries which required certificate of vaccination, demonstrates that the system can be circumvented.^23^^,^^24^

Given the presence of dengue, Zika and chikungunya in Asia, the absence of YF is the subject of much debate. Phylogenetic data indicate that YF originated in Africa and was introduced to the Americas via the slave trade out of West Africa.^25^ It has been suggested that YF had fewer historic opportunities for introduction into Asia, as Asian–African trade routes were primarily with East Africa, where YF incidence was lower.^26^^,^^27^ However, as YF spread to both the Americas and Europe, it is unlikely that the opportunity for YF introduction into Asia never occurred.^25^ Successful control of YF across Africa and the South America, through preventive vaccination campaigns or outbreak response vaccination, may have limited YF introduction into Asia during the 20th century.^1^ Conversely, dengue, chikungunya and Zika do not have licenced or effective vaccines, which has likely contributed to their global expansion.

Cross-protective immunity, due to high population exposure to dengue and other flaviviruses widespread across Asia, has also been suggested to limit YF introduction.^28^ Studies have demonstrated that although previous exposure to flaviviruses does not inhibit infection with YF, it does reduce viral load that could impede onward transmission to mosquitoes.^29–31^ Cross-immunity of flaviviruses would also explain why Zika outbreaks in Asia have been far more limited than those in the Americas.^17^^,^^32^ On the other hand, dengue and YF coexist in both Africa and South America, suggesting cross-immunity is not the only barrier. Other hypotheses include dengue virus outcompeting YF virus within the vector; competition between *A. aegypti*, the primary YF vector, and *A. albopictus* and geographical differences in vector competence or YF viral genotypes.^8^^,^^33–35^

Given the uncertainty surrounding the barriers to YF in Asia, predicting YF introductions remains a significant concern. Lack of historical introduction cannot be relied upon given the rise of urban YF, the expansion of global air travel and the rapid ecological and demographic changes that have occurred in the last 50 years, all of which increase the risk of YF introductions into Asia. Based on their interconnectivity with endemic countries, previous studies have suggested China, India, the United Arab Emirates and Saudi Arabia are at the greatest risk of YF introduction; however, the risk of autochthonous transmission is unknown.^36^^,^^37^

To address this, we used detailed origin–destination flight data from 2016 and YF incidence estimates, to predict the number of viraemic travellers capable of seeding local transmission in Asia. Next, in locations at risk of YF introduction, we used temperature-dependent reproduction number (*R*_0_) estimates and a branching process model to predict the probability of autochthonous transmission. Estimating the number of introductions capable of seeding onward transmission contributes to our understanding of the absence of YF in Asia.

## Methods

### YF introductions into Asia

To estimate the number of viraemic travellers who could potentially introduce YF into Asia, the model developed by Dorigatti *et al*.^38^ was implemented (see Supplementary Materials). The model links the flow of travellers between Asia and endemic countries, with the risk of contracting YF in the endemic country. Viraemic travellers include those that travel during their incubation or infectious periods. Data used to parameterise the model are presented in Table 1. We assumed air travel was the most likely route of international spread. We therefore quantified passenger volumes between endemic countries and Asian airports using 2016 travel data from the International Air Transport Association (IATA). The journey origin was assumed to be the passengers’ residency, and this analysis considered only the final destination (i.e. no transits or stopovers were included). All airports from countries within the United Nations Asia-Pacific regional group, along with those in Hong Kong, Macau and Taiwan, were included in this analysis.^39^ Estimates of the 2016 incidence of severe YF for endemic countries were obtained using the model developed by Garske *et al*.,^40^ which has been recently updated and extended to include South America and Africa.^41^ Reflecting estimates that 1 in 10 YF cases are severe,^42^ the incidence of severe YF was scaled to obtain an estimate of the total YF incidence in each endemic country.

**Table 1:** Parameters for the model developed by Dorigatti *et al*.,^38^ used to estimate the number of YF cases introduced into Asia.

**Endemic countries**	**Population size**	**2016 incidence estimates of YF**	**Duration of stay of international tourists (days)**
Africa			
Angola	28 813 000	12 760	3.40
Benin	10 872 000	4360	5.51
Burkina Faso	18 646 000	12 200	6.60
Burundi	10 524 000	2490	15.0
Cameroon	23 439 000	60 320	4.85
Central African Republic	4 595 000	8350	2.70
Chad	14 453 000	13 970	7.50
Congo	5 126 000	9110	2.70
Ivory Coast (Cote d’Ivoire)	23 696 000	32 040	3.00
Democratic Republic of the Congo	78 736 000	429 080	6.26
Equatorial Guinea	1 221 000	4640	3.78
Eritrea	4 955 000	750	8.40
Ethiopia	102 403 000	44 860	8.40
Gabon	1 980 000	10 660	2.70
Gambia	2 039 000	1780	3.50
Ghana	28 207 000	52 170	10.5
Guinea	12 396 000	16 250	8.88
Guinea Bissau	1 816 000	6620	22.0
Kenya	48 462 000	28 240	10.4
Liberia	4 614 000	5370	6.29
Mali	17 995 000	6700	6.00
Mauritania	4 301 000	2820	3.50
Niger	20 673 000	6500	8.00
Nigeria	185 990 000	82 480	7.00
Rwanda	11 918 000	2910	3.20
Sao Tome and Principe	200 000	40	2.70
Senegal	15 412 000	9960	3.50
Sierra Leone	7 396 000	9100	7.00
Somalia	14 318 000	6430	9.40
South Sudan	12 231 000	25 230	6.27
Sudan	39 579 000	7530	7.17
Tanzania	55 572 000	23 850	10.0
Togo	7 606 000	2580	2.00
Uganda	41 488 000	31 630	5.50
Zambia	16 591 000	4350	4.00
South America			
Argentina	43 847 000	1400	10.0
Bolivia	10 888 000	1360	19.0
Brazil	207 653 000	51 980	23.4
Colombia	48 653 000	2540	19.0
Ecuador	16 385 000	1790	8.50
French Guiana	244 000	230	2.60
Guyana	773 000	370	26.6
Panama	4 034 000	1110	8.00
Paraguay	6 725 000	660	4.30
Peru	31 774 000	3980	1.80
Suriname	558 000	390	15.0
Trinidad and Tobago	1 365 000	440	14.0
Venezuela	31 568 000	3280	11.7

The population size of each endemic country in 2016 was obtained from the World Health Organisation (WHO).^43^ The average length of stay of international tourists to each endemic country was obtained from either the World Tourism Organisation, the World Bank or the country’s national tourist website (Supplementary Table 1). We performed sensitivity analysis by increasing and decreasing the average length of stay of international tourists by 20%.

Finally, the incubation period for YF was assumed to follow a log-normal distribution with mean 4.6 days and variance 2.7 days.^44^ The infectious period was assumed to follow a normal distribution with mean 4.5 days and variance 0.6 days.^1^ Uncertainty was accounted for by sampling from these two distributions 10 000 times, giving full distributions for the number of YF introductions. Results are reported as mean and 95% confidence interval (CI). Only airports with an upper 95% CI exceeding one introduction are presented in the results section.

### Probability of autochthonous transmission

In cities served by an airport where the upper 95% CI exceeded one introduction and with evidence of an *A. aegypti* or *A. albopictus* mosquito population presence (Supplementary Table 2), we estimated the probability of autochthonous transmission. The transmission intensity in each city was quantified using *R*_0,_ the number of secondary cases produced on average by each infectious case in an entirely susceptible population. For YF, *R*_0_ is the product of the average number of infectious mosquitoes produced per infectious human (*R*_0_^HM^) and the average number of infectious humans produced per infectious mosquito (*R*_0_^MH^).^19^  *R*_0_^HM^ and *R*_0_^MH^ values were estimated for both *A. aegypti* and *A. albopictus,* separately. As temperature is a key determinant of YF transmission,^44–48^, we estimated *R*_0_^MH^ and *R*_0_^HM^ using a temperature-dependent model, following Gaythorpe *et al*.^49^ and Mordecai *et al*.^50^ (see Supplementary Materials). The mosquito biting rate and the extrinsic incubation period (EIP) were modelled using thermal response estimates (Supplementary Table 3). Temperature-dependent estimates for the EIP of *A. albopictus* were unavailable, so we scaled those of *A. aegypti* by the ratio of the point estimate recorded for *A. albopictus* (9 days at 26.7°C),^51^ divided by an estimate predicted by the temperature-dependent model recorded for *A. aegypti* at the same temperature (7.3 days at 26.7°C). Vector longevity was parameterised using estimates on *A. albopictus* and *A. aegypti* mortality from field observations, over a range of different temperatures.^45^ The temperature-dependent variables were estimated at each city’s average, minimum and maximum temperature^52^ (Table 2).

**Table 2:** Location-specific data used to estimate *R*_0_ in Asian cities predicted to be at risk of YF introduction with *A. aegypti* and/or *A. albopictus* populations.

**Country**	**City**	**Average temperature (minimum—maximum) (°C)**	**EIP (days)** ^**a**^	**Mosquito biting rate per day** ^**a**^	**Average mosquito lifespan (days)** ^**a**^	**Proportion of mosquitoes surviving EIP** ^**a**^	**Female mosquitoes per person**
*Aedes aegypti*							
India	Ahmedabad	25.19 (11.34–39.04)	9.01	0.61	16.13	0.57	0.88
Thailand	Bangkok	27.47 (20.14–34.81)	6.89	0.67	15.90	0.65	0.85
Hong Kong	Hong Kong	21.02 (11.67–30.38)	20.61	0.47	16.13	0.28	0.85^c^
Saudi Arabia	Jeddah	23.20 (7.87–38.52)	7.29	0.66	15.98	0.63	0.85^d^
Malaysia	Kuala Lumpur	26.58 (21.18–31.99)	7.57	0.65	16.02	0.62	0.82
Philippines	Manila	27.48 (21.27–33.69)	6.88	0.67	15.90	0.65	0.85^c^
Saudi Arabia	Medina	23.20 (7.87–38.52)	7.98	0.64	16.06	0.61	0.85^d^
India	Mumbai	26.55 (13.78–39.33)	7.60	0.65	16.02	0.62	0.88
Oman	Muscat	25.33 (14.44–36.22)	8.85	0.61	16.10	0.58	0.85^d^
India	New Delhi	23.89 (7.22–40.55)	10.99	0.57	16.17	0.51	0.88
Singapore	Singapore	27.08 (22.62–31.54)	7.17	0.66	15.96	0.64	0.82^c^
***Aedes albopictus***							
Thailand	Bangkok	27.47 (20.14–34.81)	8.26	0.35	43.69	0.83	2.70^c^
China	Beijing	8.50 (−12.52–29.53)	*^b^	0.00	22.00	0.00	1.97
Lebanon	Beirut	21.09 (10.44–31.74)	24.26	0.16	43.25	0.57	2.36^d^
China	Guangzhou	20.09 (8.33–31.84)	33.97	0.13	42.10	0.45	2.80
Hong Kong	Hong Kong	21.02 (11.67–30.38)	24.73	0.16	43.15	0.56	2.80^c^
Malaysia	Kuala Lumpur	26.58 (21.18–31.99)	9.09	0.33	44.27	0.81	2.70
Philippines	Manila	27.48 (21.27–33.69)	8.25	0.35	43.69	0.83	2.70^c^
India	Mumbai	26.55 (13.78–39.33)	9.12	0.33	44.27	0.81	2.70^c^
India	New Delhi	23.89 (7.22–40.55)	13.18	0.25	45.00	0.75	2.70^c^
South Korea	Seoul	10.43 (−8.22 to 29.09)	*^b^	0.00	26.52	0.00	1.97^c^
China	Shanghai	16.00 (0.77–31.23)	*^b^	0.03	36.85	0.00	1.97
Singapore	Singapore	27.08 (22.62–31.54)	8.60	0.34	43.96	0.82	2.70^c^
Japan	Tokyo	13.62 (−2.48 to 29.71)	*^b^	0.00	33.23	0.00	1.97^c^
**Data Sources**		^52^	^49^ ^,^ ^51^	^49^	^45^	^45^ ^,^ ^49–51^	^53–57^

^a^Temperature-dependent variable.

^b^Average temperature at this location is outside of the temperature range for the EIP. Therefore, parasite development within the mosquito will not occur and this location is unsuitable for transmission at the average temperature.

^c^Estimate from nearby country.

^d^No local estimate available. Average of all the studies of either *A. aegypti* or *A. albopictus* density that we identified across Asia.

Where possible, we obtained local estimates for the number of female *A. aegypti* and *A. albopictus* per person.^53–57^ Where these data were unavailable, an alternative estimate of *Aedes* mosquito density or an estimate from the nearest location were used (Table 2). All other parameters were assumed to be constant across spatial units. The effective transmission rates between vector and host were taken from a study, which assessed the YF infection and transmission rates of Asian *A. aegypti* and *A. albopictus.*^9^ Thus, the effective transmission rate from human to *A. aegypti* was set at 0.8, and the effective transmission rate from *A. aegypti* to human was set at 0.24. For *A. albopictus* these values were 0.26 and 0.13, respectively. We performed sensitivity analyses on the assumed number of female *Aedes* mosquitoes per person and their competency (see Supplementary Materials).

We assumed independent introductions of infectious humans and quantified the outbreak probability utilising the methodology developed by Johansson *et al*.^19^ and adapted by Luo *et al*.^58^ This model uses a branching process and draws from negative binomial offspring distributions with means *R*_0_^MH^ and *R*_0_^HM^ and dispersion parameter $k$ (see Supplementary Materials). The dispersion parameter controls individual heterogeneity in infectiousness and was assumed to be high ($k$ = 0.1), as estimated for other vector-borne diseases.^59–61^ We performed sensitivity analyses to evaluate the role of individual-level heterogeneity in infectiousness ($k$) and population immunity (varied from 0 to 100%) on the probability of autochthonous transmission.

Analyses were performed in R version 3.6.1 and using the *Epiflows* package.^62^

## Results

**Figure 1:**
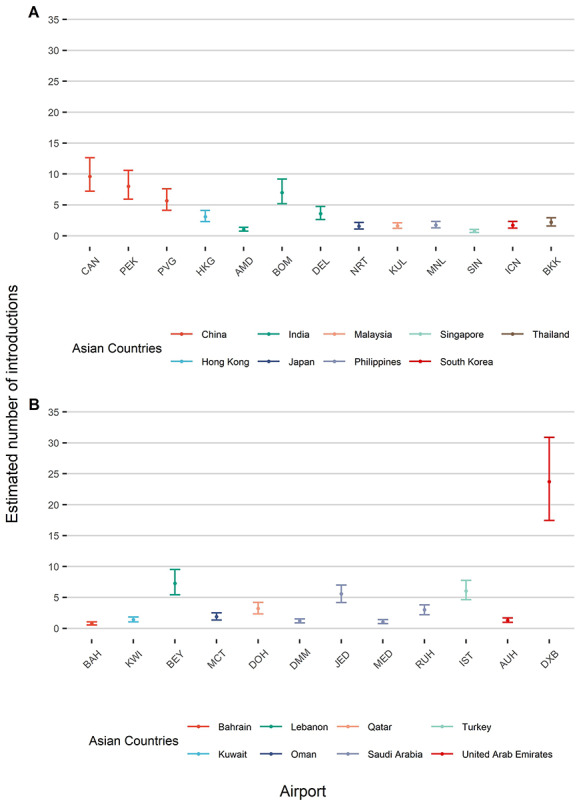
Mean (point) and 95% CIs (bars) of the total predicted number of introductions of YF into (A) airports in South and East Asia and (B) airports in West Asia and the Middle East. Introductions presented are aggregated from all endemic countries. Only airports with an upper 95% confidence limit greater than one introduction are shown. CAN: Guangzhou Baiyun International Airport, PEK: Beijing Capital International Airport, PVG: Shanghai Pudong International Airport, HKG: Hong Kong International Airport, AMD: Sardar Vallabhbhai Patel International Airport; BOM: Chhatrapati Shivaji International Airport, DEL: Indira Gandhi International Airport, NRT: Narita International Airport, KUL: Kuala Lumpur International Airport, MNL: Ninoy Aquino International Airport, SIN: Singapore Changi Airport, ICN: Incheon International Airport, BKK: Suvarnabhumi Airport, BAH: Bahrain International Airport, KWI: Kuwait International Airport, BEY: Beirut–Rafic Hariri International Airport, MCT: Muscat International Airport, DOH: Hamad International Airport, DMM: King Fahd International Airport, JED: King Abdulaziz International Airport, MED: Prince Mohammad bin Abdulaziz International Airport, RUH: King Khalid International Airport, IST: Istanbul Airport, AUH: Abu Dhabi International Airport, DXB: Dubai International Airport

We estimated that 25 airports in Asia were at risk of YF introductions during 2016, defined as having an upper 95% CI exceeding one introduction (Figure 1). These estimates refer to the overall number of introductions aggregated from all endemic countries. China, India, Saudi Arabia and the United Arab Emirates all had multiple airports predicted to be at risk of at least one YF introduction. Sensitivity analyis on the duration of stay did not change the risk of YF introduction predicted into each city (not shown).

**Figure 2:**
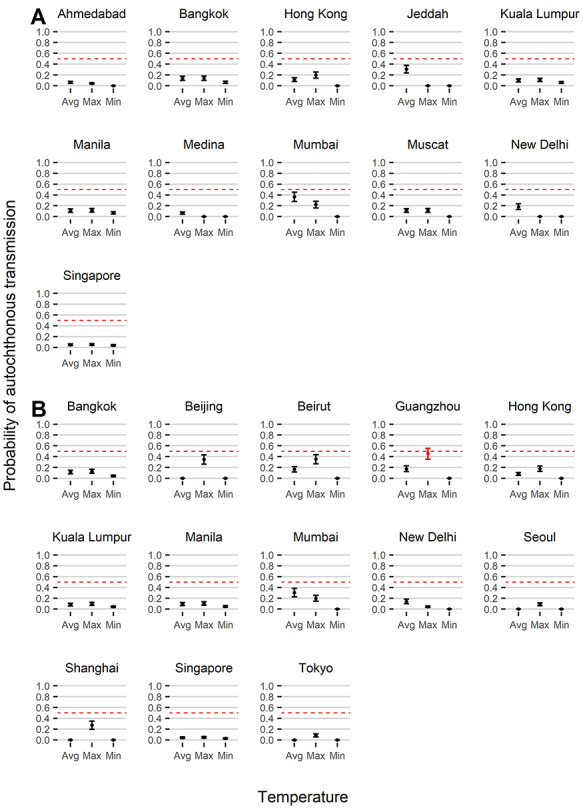
Mean (point) and 95% CIs (bars) of the predicted probabilities of autochthonous transmission in Asian cities, assuming transmission by (A) *A. aegypti* and (B) *A. albopictus*, given the independent introduction of at least one infectious individual. Probabilities are estimated at the average, minimum and maximum temperature for each location. Probabilities in red indicate an upper 95% probability of autochthonous transmission exceeding 0.5, denoted by the dashed red line

Next, we estimated *R*_0_ values for cities with evidence of a competent mosquito population (Supplementary Figure 1). In cities where *A. albopictus* and *A. aegypti* are both present, we estimated two independent *R*_0_ values. The *R*_0_ estimate and number of introductions for each city were then used to predict the outbreak probability. Figure 2A shows that the risk of autochthonous transmission is <50% across all temperatures for cities in Asia with an *A. aegypti* population. Similarly, Figure 2B shows the risk of autochthonous transmission is <50% for Asian cities with *A. albopictus* populations, apart from Guangzhou at its maximum temperature, where the upper bound of the 95% CI exceeds 50%.

**Figure 3:**
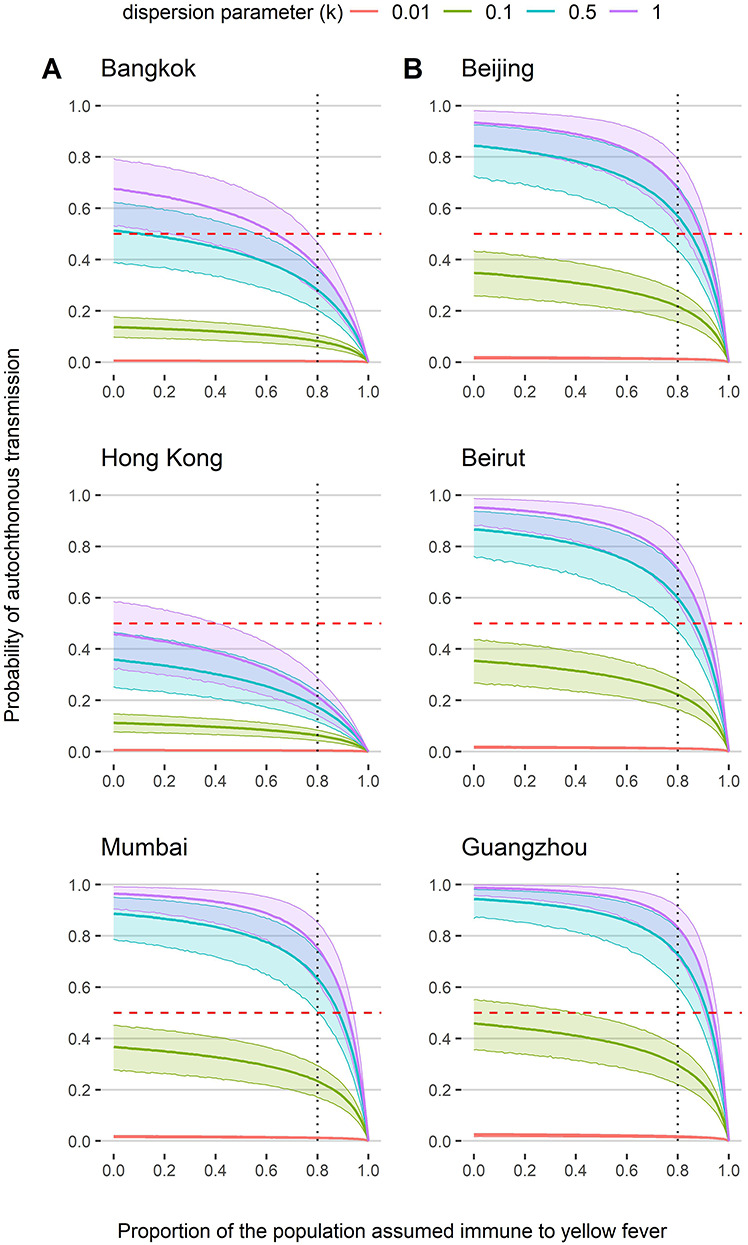
Probability of autochthonous transmission across increasing levels of population immunity at (A) the average temperature in Bangkok, Hong Kong and Mumbai assuming transmission by *A. aegypti* and (B) the maximum temperature in Beijing, Beirut and Guangzhou assuming transmission by *A. albopictus*. Also presented are probabilities at different values of the dispersion parameter *k*, where purple = 1, blue = 0.5, green = 0.1(baseline value) and red = 0.01. Thick lines are the mean and shaded areas are the 95% CIs. Horizontal dashed red line represents the point at which the probability of autochthonous transmission is <0.5. Vertical dashed line denotes 80% population immunity recommended by the WHO to stop local transmission in endemic countries

Increasing individual heterogeneity in infectiousness (which is obtained by decreasing the dispersion parameter $k$ to 0.01) reduced the risk of onward transmission to near 0 in all cities (Figure 3). Conversely, only high levels of population immunity had a large reduction on the risk of transmission (Figure 3). For instance, in Mumbai, assuming achievement of the WHO recommended 80% vaccination coverage is estimated to reduce the probability of transmission by 36%, whereas a vaccination coverage of 95% would reduce transmission by 68%. Sensitivity analysis found that the effective transmission rate from mosquitoes to humans had a minimal effect on the risk of transmission (Supplementary Figure 2). Finally, in cities where the probability of autochthonous transmission was very low, the number of female mosquitoes per person had a limited effect on this risk. Conversely, cities predicted to have a higher probability of transmission were more sensitive to assumptions about the number of female mosquitoes per person.

## Discussion

Quantifying the risk of YF introduction by viraemic travellers who have the potential to propagate local transmission is critical for assessing the risk of global YF spread and can provide useful information when additional control measures are considered.^63^ We identified 25 cities in Asia at risk of YF introduction from endemic countries during 2016. However, except for Guangzhou at its maximum temperature, the outbreak probability across Asia was low (i.e. the 95% CI of the outbreak probability did not exceed 50%).

We predicted introductions into multiple airports in China, in accordance with the observed case introductions in 2016.^7^ Additionally, case introductions were predicted into multiple airports in India, the United Arab Emirates and Saudi Arabia, not observed in 2016, but in agreement with a study analysing travel patterns between YF endemic and at-risk countries.^36^ Brent *et al*.^36^ reported the total number of passengers flying between endemic countries and countries at risk for YF (defined by their suitability for dengue), as a proxy for the risk of YF introduction. In contrast, we use estimates of the number of travellers provided by IATA, to predict the expected number of viraemic travellers introduced, which was then used to also quantify the probability of autochthonous transmission. As we predicted a low probability of autochthonous transmission, introductions are likely to go undetected, given the high proportion of cases that have no or non-specific symptoms.^1^ This may explain the absence of recorded case introductions into Asia, outside of China.

Other assessments of YF in Asia highlight South and Southeast Asia as suitable for transmission.^64^^,^^65^ In agreement with this, we predicted transmission intensity to be highest in India, Malaysia, the Philippines, Singapore, Hong Kong and Thailand, with temperature-dependent *R*_0_ estimates close to *R*_0_ values seen during urban outbreaks in endemic countries.^66^^,^^67^ Despite this, we found low support for autochthonous transmission due to an estimated low number of case introductions. Although flight data were unavailable beyond 2016, tourist arrivals in Asia are estimated to increase on average by 5% annually,^68^ so the risk of introduction can also be expected to have increased in recent years. Equally, introductions are likely to increase following rises in YF incidence in endemic countries, as seen with the Angola outbreak.^7^

Higher probabilities of autochthonous transmission in China and Lebanon at warmer temperatures suggest that the potential for YF transmission by *A. albopictus* is seasonal. At the maximum temperature we predicted a risk of autochthonous transmission in Guangzhou, which regularly experiences dengue outbreaks in the summer, supporting the continued use of seasonal vector control measures.^69^ The YF case introductions into China in 2016 occurred in March and April, when *A. albopictus* populations were reduced, and could explain the absence of onward transmission.^70^ However, climate change is expanding the habitat of *Aedes* mosquitoes and extending periods of transmission suitability, indicating the risk will increase.^71^ Cities predicted to be at risk of future establishment of *Aedes* mosquitoes should also be monitored.^72^ For instance, in Dubai a high number of introductions were predicted, and dengue outbreaks occur in neighbouring Saudi Arabia and Oman.^73^^,^^74^

Alongside insufficient introductions and seasonality, we found heterogeneity in individual-level infectiousness to be a limiting factor to YF in Asia, which may arise through several mechanisms including asymptomatic infection and human, virus or mosquito genetics.^61^^,^^75^ Although cross-immunity with related flaviviruses could limit YF transmission in Asia, a high proportion of the population would need to develop immunity to significantly reduce the probability of autochthonous transmission.

We estimate that reduced vector competency is unlikely to be a substantial barrier to YF transmission in Asia, as the effective transmission rate from mosquito to humans had a limited effect on the probability of autochthonous transmission. Furthermore, the *R*_0_ estimates and corresponding probabilities of autochthonous transmission for *A. albopictus* were similar to *A. aegypti*, despite much lower effective transmission rates. This may be explained by *A. albopictus’* longer lifespan and higher probability of surviving the EIP, which increases its competency as a YF vector, as has previously been suggested for dengue.^76^ It is also thought that adaptation of chikungunya virus to *A. albopictus* may have facilitated global spread and the recent chikungunya outbreaks in Asia.^15^ Thus, *A. albopictus* has the potential to be an active YF vector and its widespread distribution is unlikely to be a major barrier to YF establishment in Asia.^35^

Our results suggest that a limited number of case introductions in 2016 was the primary reason for the low predicted probability of autochthonous transmission in Asia. However, we also find that a number of other factors can reduce the probability of autochthonous transmission, and these could have an additive effect in preventing the establishment of YF in Asia. This is in agreement with the current literature, which suggests that multiple societal, biological and environmental factors likely contribute to the absence of YF in Asia.^8^^,^^27^^,^^77^

This study is not without limitations. When estimating case introductions, we assumed that the origin of each journey was the passengers’ residency and that the final travel destinations in the data set were the actual final destinations, rather than stopovers. Thus, the risk of introduction and the probability of autochthonous transmission may be overestimated for hubs, which act as transit even though appear as final destinations in the data set. Only air travel was accounted for, whereas introductions could occur via sea and land, especially between the Middle East and northeast Africa.^78^ Our estimates do not account for medium- or long-term migration, for instance due to work.^37^^,^^79^ Including migrants, along with tourists, in the model would increase the estimated number of importations but in the absence of detailed data on the migrant population between endemic countries and Asia and on their length of stay, it is hard to quantify the impact of migration on the risk of YF introduction.

We assumed force of infection was homogeneous within each endemic country, overlooking geographic variation, especially in holoendemic countries. Infection risk is also generally higher for residents, although some tourists may be high risk owing to lower immunity (through lack of natural exposure or vaccination) or due to behavioural risks, like ecotourism, increasing exposure.^23^^,^^25^^,^^77^ Force of infection was assumed to be constant over time and does not capture the time-varying intensity of transmission during an outbreak, nor the seasonality in travel patterns. Finally, the socioeconomic characteristics of at-risk countries were not accounted for, like those with health infrastructures weakened by conflict.^80^

Cities with higher probabilities of transmission were sensitive to assumptions made about mosquito densities, indicating the risk could have been either under- or overestimated. There are limited data available on mosquito densities and those that are available are heterogeneous due to variability in the density indicator, season, timing (i.e. in response to an outbreak), trap location and trap type.^53–57^^,^^73^^,^^81–83^ Data on vector density at a higher spatial resolution using standardised methodology would provide a more comprehensive analysis. Alternatively, Massad *et al*.^84^^,^^85^ previously used the incidence of dengue to estimate *A. aegypti* density and the probability of urban YF outbreaks, following the theoretical introduction of an infected traveller into Brazil. In future analyses, it would be interesting to apply this method to assess the risk of YF in Asia, considering our vector density data limitations. However, if dengue is a barrier to YF in Asia, either through cross-immunity or viral competition, data on dengue incidence may overestimate the risk in dengue hyperendemic areas and underestimate the risk in areas with competent mosquito populations but less dengue transmission, such as Southern China.^8^

We also assumed exclusively urban transmission and parameterised the model using *A. aegypti* and *A. albopictus* data. However, the exact contribution of each species to YF transmission is unknown, even in countries endemic for YF, so for cities infested by both mosquitoes we have reported two independent estimates of the probability of autochthonous transmission. Finally, we did not attempt to distinguish between different YF viral genotypes and vector competence was assessed solely using a West African genotype of the YF virus.^9^

The study has several strengths. The methods applied in this study have previously been used to predict Zika introductions into the USA and agreed with the observed number of introductions.^58^ Equally, temperature-dependent *R*_0_ estimates have previously been shown to be important predictors of the probability of autochthonous transmission of vector-borne diseases.^50^ Moreover, the risk of autochthonous transmission quantified here is dependent on both the number of introductions and on the local intensity of transmission, which provides critical information to public health officials beyond YF suitability and travel patterns.

We show that despite the rise of air travel, limited YF introductions capable of seeding onwards transmission continue to act as a barrier to YF in Asia. This supports ongoing surveillance, in order to monitor the future number of introductions, as increases in travel volumes or in the incidence of YF in endemic countries may increase the outbreak probability in Asia. Surveillance of YF presents challenges, owing to YF’s broad clinical spectrum and cross-reactivity with other flaviviruses. Therefore, the modelling framework in this study is a useful tool to monitor the risk of YF in Asia, by estimating future YF case introductions. Our results also suggest that ensuring travellers from and to YF endemic countries are vaccinated would be the most effective control measure to minimise the outbreak risk in Asia.

Should YF introduction occur, the large volumes of intraregional travel within Asia would facilitate the spread of YF further, as seen with dengue transmission.^86^^,^^87^ This analysis also suggests that vaccination coverages >80% would be required to significantly reduce the probability of autochthonous transmission, which may pose challenges given the current shortage of vaccine doses available.

This study quantifies the risk of YF in Asia using the limited data available and tests whether the expected number of YF introductions would have supported local transmission. We found a limited number of YF introductions producing a low probability of autochthonous transmission in 2016, suggesting that a limited number of introductions over past years may have historically prevented YF establishment in Asia. Although the model simplifies the complexity of YF transmission, it allows us to produce data-driven estimates of the expected number of introductions and of the probability of YF establishment in Asia, which can serve as a useful tool to monitor the risk of YF introduction. Given the global challenges in YF surveillance, the methods presented in this study can help identify the areas at risk of YF introduction and inform decisions to minimise the outbreak potential to reduce the global burden of YF in the future.

## Supplementary Material

YF_in_Asia_supplementary_material_taab015

## Code availability

The code and data used to produce all results are available at: https://github.com/bnc19/YF_in_Asia
