# Supplementary material for: Yellow fever in Asia–a risk analysis
Source: J Travel Med. 2021 Jan 28;28(3):taab015. doi: 10.1093/jtm/taab015 (PMC8045179; doi:10.1093/jtm/taab015)
Supplement: YF_in_Asia_supplementary_material_taab015 [file yf_in_asia_supplementary_material_taab015.docx]

# **Supplementary material**

**Yellow fever in Asia – a risk analysis**

Bethan Cracknell Daniels ^1^, Katy Gaythorpe^1^, Natsuko Imai^1^, Ilaria Dorigatti^1^

^1^ MRC GIDA and J-IDEA: MRC Centre for Global Infectious Disease Analysis; and the Abdul Latif Jameel Institute for Disease and Emergency Analytics (J-IDEA), School of Public Health, Imperial College London.

### **Estimating the number of yellow fever introductions into Asia**

Here, we outline the model of Dorigatti et al. ^1^, used to predict the number of introductions of yellow fever (YF) from endemic countries into Asia. The total number of introductions includes exportations and importations. An exportation is an individual from an endemic region who visits Asia whilst incubating or infectious. An importation is an individual from Asia who travels to an endemic country, contracts YF and then returns to Asia whilst incubating or infectious.

Exportations:

Let $A$ denote the Asian location at risk of YF introduction. Let $D$ denote the endemic country. The total number of expected exportations from $D$ to $A$ is therefore $E_{DA}$ and is the product of the per capita probability of an individual from $D$ travelling to $A$ ($P_{DA}$), the cumulative number of YF infections in location $D$ during 2016 ($C_{D}$) and the probability of an individual travelling during 2016 whilst infectious ($P_{i}$):

| $E_{DA}= P_{DA} . C_{D} . P_{i}$ | [1.1] |
| --- | --- |

The per capita probability of an individual from $D$ travelling to $A$ ($P_{DA}$) is in turn derived from the number of travellers leaving $D$ to travel to $A$ in 2016 ($T_{DA}$) and the population of $D$ (${pop}_{D}$):

| $P_{DA}= \frac{T_{DA}}{{pop}_{D}}$ | [1.2] |
| --- | --- |

The probability of an individual travelling whilst infectious during 2016 ($P_{i}$) includes those travelling during either the incubation ($D_{E}$) or the infectious period ($D_{I}$). As 2016 was a leap year, the probability of travelling whilst viraemic is derived by sampling from these distributions and dividing by 366 days:

| $P_{i}=minimum \left( \frac{D_{E} + D_{I}}{{366}_{days}}, 1 \right)$ | [1.3] |
| --- | --- |

Importations:

Let $I_{AD}$ be the total expected number of importations into $A$ from $D$*.* $I_{AD}$ is therefore the product of the number of travellers visiting $D$ from their home location $A$ during 2016 ($T_{AD}$), their per capita risk of infection whilst visiting $D$ ($\lambda_{D}$) and the probability of them returning to $A$ whilst incubating or infectious ($P_{r}$):

| $I_{AD}=T_{AD} . \lambda_{D} . P_{r}$ | [1.4] |
| --- | --- |

In turn, $\lambda_{D}$ is calculated as:

| $\lambda_{D}= \frac{C_{D} . L_{D}}{{pop}_{D} . {366}_{days}}$ | [1.5] |
| --- | --- |

Where $L_{D}$ is the average duration of stay of an international tourist in endemic country $D$ and $C_{D}$and ${pop}_{D}$ are defined as above. Finally, the probability of returning to $A$ whilst incubating or infectious is:

| $P_{r}=minimum \left( \frac{D_{E}+ D_{I}}{L_{D}}, 1 \right)$ | [1.6] |
| --- | --- |

Total number of introductions:

The total number of introductions into $A$ from $D$ is given as the sum of the number of exportations and importations:

| ${Intro}_{AD}=E_{DA}+ I_{AD}$ | [1.7] |
| --- | --- |

**Temperature-dependent model of the reproduction number**

The risk of autochthonous transmission for vector-borne diseases depends on local ecological conditions, which can be quantified through the reproduction number (R_0_)_._ R_0_ is the product of the average number of infectious mosquitos produced per infectious human (R_0_^HM^) and the average number of infectious humans produced per infectious mosquito (R_0_^MH^) ^2^.

R_0_^HM^ is in turn the product of the number of female mosquitos per person ($\delta$), the average mosquito biting rate ($\alpha$), the effective transmission rate from human to mosquito ($\beta_{HM}$), the probability of the mosquito surviving the extrinsic incubation period ($p_{surv}$) and the mean duration of the human infectious period ($D_{I}$):

| $R_{0}^{\mathrm{HM}}= \delta. \alpha. \beta_{HM} . p_{surv} . D_{I}$ | [2.1] |
| --- | --- |

The probability that the mosquito survives the extrinsic incubation period ($p$) and may therefore go on to infect a person ($p_{surv}$) is derived by assuming an exponential distribution, where $L$ is the average life span of the mosquito:

| $p_{surv}=e (\frac{- p}{L})$ | [2.2] |
| --- | --- |

R_0_^MH^ is the product of the average mosquito biting rate ($\alpha$), the effective transmission rate from mosquito to human ($\beta_{MH}$) and the average life span of the mosquito ($L$):

| $R_{0}^{\mathrm{MH}}= \alpha. \beta_{MH} . L$ | [2.3] |
| --- | --- |

In this analysis, R_0_^MH^ and R_0_^HM^ were estimated separately for two species of mosquito with the potential to transmit YF, *Aedes (Ae.) aegypti* and *Ae. albopictus*, using a temperature-dependent model ^3,4^. The mosquito biting rate ($a$) and the extrinsic incubation period ($p$) were modelled as Briere functions of temperature:

| $a\left( T_{A} \right)={a_{c} . T_{A} . \left( T_{A} -a_{T0} \right) . (a_{Tm}-T_{A})}^{0.5}$ | [2.4] |
| --- | --- |

| $p\left( T_{A} \right)= \frac{1}{{p_{c} . T_{A} . \left( T_{A} - p_{T0} \right) . (p_{Tm}- T_{A})}^{0.5}}$ | [2.5] |
| --- | --- |

Where $T_{A}$ denotes the mean temperature of each Asian location. The subscripts $T0$ and $Tm$ are the thermal minimum and maximum values and the subscript $c$ is a positive rate constant. For *Ae, aegypti*, the mosquito biting rate ($a$) and the extrinsic incubation period ($p$) at the minimum and maximum temperatures were fitted using experimental estimates from Gaythorpe et al. ^3^, whilst the mosquito biting rate ($a$) for *Ae. albopictus* was fitted using data from Mordecai et al ^4^ (**Supplementary Table 3**). All variables were limited to be positive.

Full distributions of R_0_^HM^ and R_0_^MH^, and therefore R_0,_ were obtained by sampling from the duration of the human infectious period ($D_{I}$) 10,000 times.

**Probability of autochthonous transmission**

To estimate the risk of local transmission, the methodology of Johansson et al. ^2^ adapted by Luo et al. ^5^ was implemented. This method uses a branching process to estimate the offspring distribution at each generation. R_0_^HM^ and R_0_^MH^ are assigned the subscript $A$ for each at-risk location. Assuming the introduction of one infectious human, the probability of a given number of mosquitos becoming infected can be estimated by drawing from a negative binomial distribution with mean R_0_^HM^ and dispersion parameter $k$, described by $g_{M, A}\left( s \right)$:

| $g_{M, A}\left( s \right)={[1+ \frac{R_{0, A}^{HM}}{k}\left( 1-s \right)]}^{-k}$ | [2.7] |
| --- | --- |

The probability of a mosquito infecting a given number of humans is similarly described by $g_{H, A}\left( s \right)$:

| $g_{H, A}\left( s \right)={[1+ \frac{R_{0, A}^{MH}}{k}\left( 1-s \right)]}^{-k}$ | [2.8] |
| --- | --- |

The probability of immediate extinction (i.e., no onward transmission) is found by analysing the value $g\left( 0 \right)$. The composite function of these events thus gives the probability that the introduction of an infectious human results in no autochthonous transmission:

| $g_{M, A}\left( g_{H, A}\left( 0 \right) \right)= {[1+ \frac{R_{0, A}^{\mathrm{HV}}}{k} \left( 1- g_{H, A}\left( 0 \right) \right)]}^{-k}$ | [2.9] |
| --- | --- |

The probability of autochthonous transmission is thus 1 minus the probability of no transmission:

| $P_{auto}(A){= 1- g}_{M, A}\left( g_{H, A}\left( 0 \right) \right)$ | [2.10] |
| --- | --- |

Finally, given the introduction of multiple independent infectious humans ($Intro$), the probability of autochthonous transmission at each at-risk location can be given as:

| $P_{auto}(A){{= 1- g}_{M, A}\left( g_{H, A}\left( 0 \right) \right)}^{Intro}$ | [2.11] |
| --- | --- |

### **Sensitivity analysis**

We performed sensitivity analyses to assess the impact of changes in the assumed number of female mosquitoes per person ($\delta$) on the probability of autochthonous transmission. $\delta$ was set at 0.1, 0.45, 0.85, 2, 4 and 5, in line with the range of estimates we found for the number of *Ae*. mosquitoes per person across Asia ^6-14^. The effective transmission rate from mosquito to human ($\beta_{MH}$) was set at 0.24 for *Ae. aegypti* and 0.13 for *Ae. albopictus* ^15^. As competency of Asian mosquitos could be a barrier to YF establishment, sensitivity analysis was performed by ranging the value of $\beta_{MH}$ from 0 to 1, in intervals of 0.1. Equally, population immunity was increased from 0 – 100% to explore the role of cross-immunity in limiting the introduction of YF into Asia. To this end, R_0_^MH^ was replaced with R_eff_^MH^, the effective number of infectious humans produced per infectious mosquito. R_eff_^MH^ can be derived by adjusting R_0_^MH^ with the proportion of the population who possess immunity:

| $R_{eff}^{MH}= R_{0}^{MH} . (1 -{pop}_{immunity})$ | [2.12] |
| --- | --- |

Finally, sensitivity analysis of the value of the dispersion parameter $k$ was implemented to assess the role of individual heterogeneity in transmission on the probability of local transmission. The values of $k$ evaluated were 0.01, 0.1 (baseline value), 0.5 and 1 ^16^.

**Supplementary Tables**

**Supplementary Table 1:** Data used estimate the average duration of stay of an international visitor to each endemic country. Data was collected from either the World Tourism Organisation, the World Bank Group, or the national tourist website for each country. Where this data could not be found, the average was taken from neighbouring countries (those that share a border).

| **Country** | **Duration of Stay (days)** | **Source** |
| --- | --- | --- |
| Angola | 3.40 | ^17^ |
| Benin | 5.51 | ^18^ |
| Burkina Faso | 6.60 | ^19^ |
| Burundi | 15.0 | ^20^ |
| Cameroon | 4.85 | ^21^ |
| Central African Republic | 2.70 | Average of neighbouring countries |
| Chad | 7.50 | Average of neighbouring countries |
| Republic of Congo | 2.70 | ^22^ |
| Ivory Coast (Cote d'Ivoire) | 3.00 | ^23^ |
| Democratic Republic of the Congo | 6.26 | Average of neighbouring countries |
| Equatorial Guinea | 3.78 | Average of neighbouring countries |
| Eritrea | 8.40 | Average of neighbouring countries |
| Ethiopia | 8.40 | ^24^ |
| Gabon | 2.70 | Average of neighbouring countries |
| **Country** | **Duration of Stay (days)** | **Source** |
| Gambia | 3.50 | Average of neighbouring countries |
| Ghana | 10.5 | ^25^ |
| Guinea | 8.88 | Average of neighbouring countries |
| Guinea Bissau | 22.0 | ^26^ |
| Kenya | 10.4 | ^27^ |
| Liberia | 6.29 | Average of neighbouring countries |
| Mali | 6.00 | Average of neighbouring countries |
| Mauritania | 3.50 | Average of neighbouring countries |
| Niger | 8.00 | ^28^ |
| Nigeria | 7.00 | ^29^ |
| Rwanda | 3.20 | ^30^ |
| Sao Tome and Principe | 2.70 | Average of neighbouring countries |
| Senegal | 3.50 | ^31^ |
| Sierra Leone | 7.00 | ^32^ |
| Somalia | 9.40 | Average of neighbouring countries |
| South Sudan | 6.27 | Average of neighbouring countries |
| Sudan | 7.17 | Average of neighbouring countries |
| Tanzania | 10.0 | ^33^ |
| Togo | 2.00 | ^34^ |
| Uganda | 5.50 | ^35^ |
| Zambia | 4.00 | ^36^ |
| Argentina | 10.0 | ^37^ |
| Bolivia | 19.0 | ^38^ |
| **Country** | **Duration of Stay (days)** | **Source** |
| Brazil | 23.4 | ^39^ |
| Colombia | 19.0 | ^40^ |
| Ecuador | 8.50 | ^41^ |
| French Guiana | 2.60 | ^42^ |
| Guyana | 26.6 | ^43^ |
| Panama | 8.00 | ^44^ |
| Paraguay | 4.30 | ^45^ |
| Peru | 1.80 | ^46^ |
| Suriname | 15.0 | ^47^ |
| Trinidad and Tobago | 14.0 | ^48^ |
| Venezuela | 11.7 | ^49^ |

**Supplementary Table 2:** Evidence of *Aedes aegypti* and *Aedes albopictus* mosquito populations and reports of dengue, chikungunya and Zika infections, in cities in Asia predicted to be at risk of least one yellow fever introduction in 2016. UAE: United Arab Emirates.

| **City (country)** | ***Aedes aegypti* occurrence** | ***Aedes albopictus* occurrence** | **Dengue** | **Chikungunya** | **Zika** |
| --- | --- | --- | --- | --- | --- |
| Abu Dhabi  (UAE) | No | No | No | No | No |
| Ahmedabad  (India) | Occurrence recorded  ^50^ | No | Endemic  ^51^ | Outbreaks recorded ^52^ | Autochthonous transmission recorded ^53^ |
| Bangkok  (Thailand) | Occurrence recorded  ^50^ | Occurrence recorded  ^50^ | Endemic  ^51^ | Outbreaks recorded ^54^ | Outbreaks recorded ^55^ |
| Beijing  (China) | No | Occurrence recorded  ^50^ | Case introductions ^56^ | No | No |
| Beirut  (Lebanon) | No | Occurrence recorded  ^57^ | Autochthonous transmission recorded ^58^ | No | No |
| Dammam  (Saudi Arabia) | No | No | No | No | No |
| Doha  (Qatar) | No | No | No | No | No |
| Dubai  (UAE) | No | No | No | No | No |
| Guangzhou  (China) | No | Occurrence recorded  ^50^ | Outbreaks recorded  ^59,60^ | Outbreak recorded ^61^ | Case introductions ^62^ |
| Hong Kong | Occurrence recorded  ^50^ | Occurrence recorded  ^50^ | Endemic  ^51^ | Case introductions ^63^ | Case introductions ^55^ |
| Istanbul  (Turkey) | No | No | No | No | No |
| Jeddah  (Saudi Arabia) | Occurrence recorded  ^57^ | No | Outbreaks recorded  ^64^ | Outbreak recorded ^65^ | No |
| Kuala Lumpur  (Malaysia) | Occurrence recorded  ^50^ | Occurrence recorded  ^50^ | Endemic  ^51^ | Outbreaks recorded ^54^ | Autochthonous transmission recorded ^55^ |
| **City (country)** | ***Aedes aegypti* occurrence** | ***Aedes albopictus* occurrence** | **Dengue** | **Chikungunya** | **Zika** |
| Kuwait City  (Kuwait) | No | No | Positive seroprevalence study  ^64^ | No | No |
| Manama  (Bahrain) | No | No | No | No | No |
| Manila  (Philippines) | Occurrence recorded  ^50^ | Occurrence recorded  ^50^ | Endemic  ^51^ | Outbreaks recorded  ^54^ | Autochthonous transmission recorded ^55^ |
| Medina  (Saudi Arabia) | Occurrence recorded  ^57^ | No | No | No | No |
| Mumbai  (India) | Occurrence recorded  ^50^ | Occurrence recorded  ^50^ | Endemic  ^51^ | Outbreaks recorded ^52^ | No |
| Muscat  (Oman) | Occurrence recorded  ^14^ | No | Outbreak recorded  ^14^ | No | No |
| New Delhi  (India) | Occurrence recorded  ^50^ | Occurrence recorded  ^50^ | Endemic  ^51^ | Outbreaks recorded ^52^ | No |
| Riyadh  (Saudi Arabia) | No | No | No | No | No |
| Seoul  (South Korea) | No | Occurrence recorded  ^50^ | Case introductions ^66^ | No | No |
| Shanghai  (China) | No | Occurrence recorded  ^50^ | Autochthonous transmission recorded ^56^ | No | No |
| Singapore | Occurrence recorded  ^50^ | Occurrence recorded  ^50^ | Endemic  ^51^ | Outbreaks recorded ^54^ | Outbreak recorded ^55^ |
| Tokyo  (Japan) | No | Occurrence recorded  ^50^ | Outbreaks recorded ^67^ | No | Case introductions ^55^ |

**Supplementary Table 3:** Mean parameter values used to estimate the temperature-dependent biting rate and EIP. T0 and TM are the minimum and maximum values for each variable respectively and c is a positive rate constant. The temperature-dependent variables are fitted to experimental data based on Aedes aegypti and Aedes albopictus laboratory studies. EIP: Extrinsic incubation period.

| **Parameter** | ***Aedes aegypti* biting rate** | ***Aedes albopictus* biting rate** | ***Aedes aegypti* yellow fever inverse EIP** |
| --- | --- | --- | --- |
| T0 | 2.9285 | 10.25 | 17.6724 |
| TM | 40.1368 | 38.32 | 42.1075 |
| c | 0.0003 | 0.00019 | 0.0001 |
| **Source** | **^3^** | **^4^** | **^3^** |

# **Supplementary Figures**

**
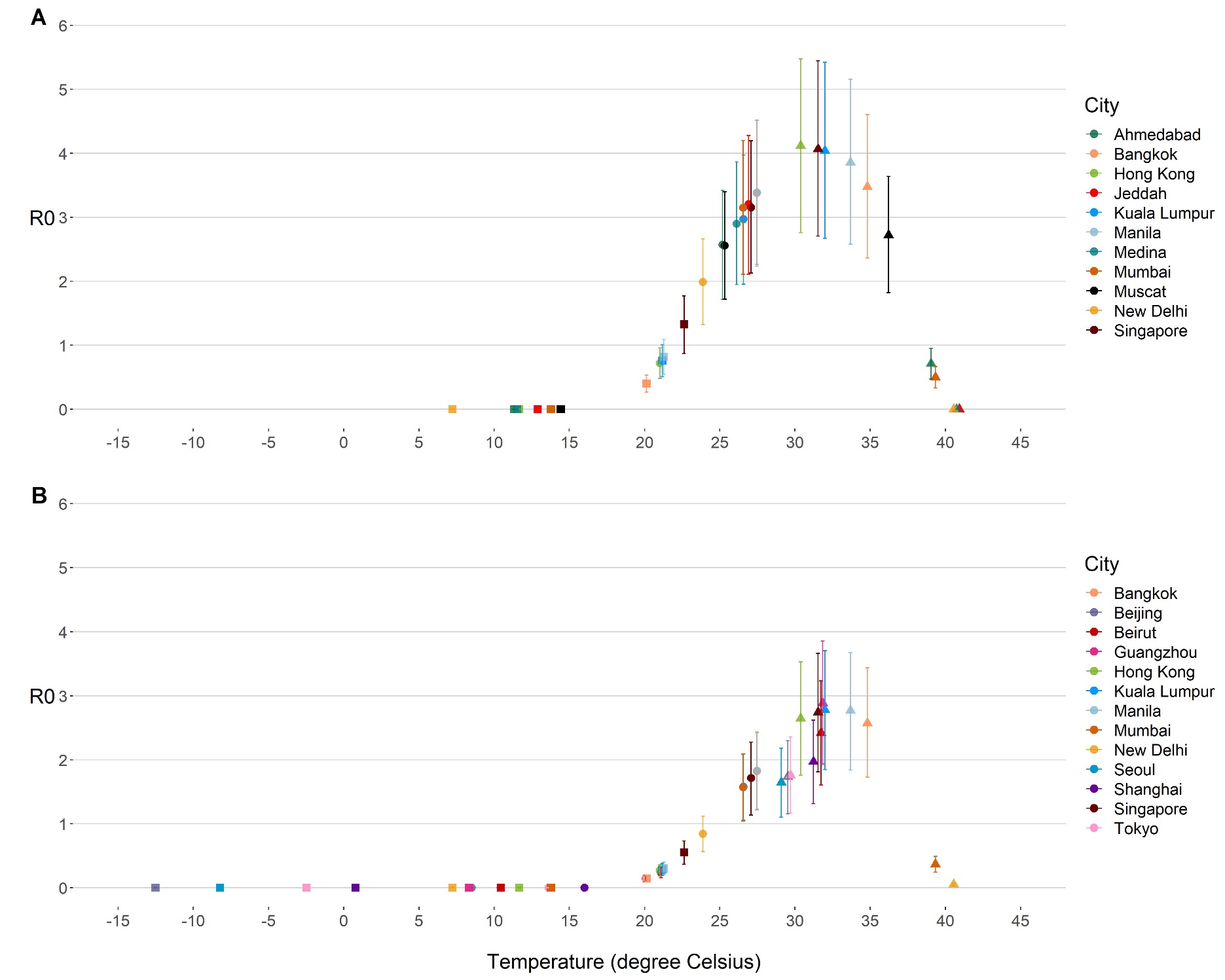
**

**Supplementary Figure 1:** Mean (point) and 95% confidence intervals (bars) of temperature-dependent R_0_ estimates in cities predicted to be at risk of yellow fever introduction, with evidence of competent mosquito populations. **A)** Transmission attributable to Aedes aegypti and **B)** transmission attributable to Aedes albopictus. Shapes denote the average, minimum and maximum temperature of each city. Temperatures are in degree Celsius (°C). R_0_: reproduction number.

###
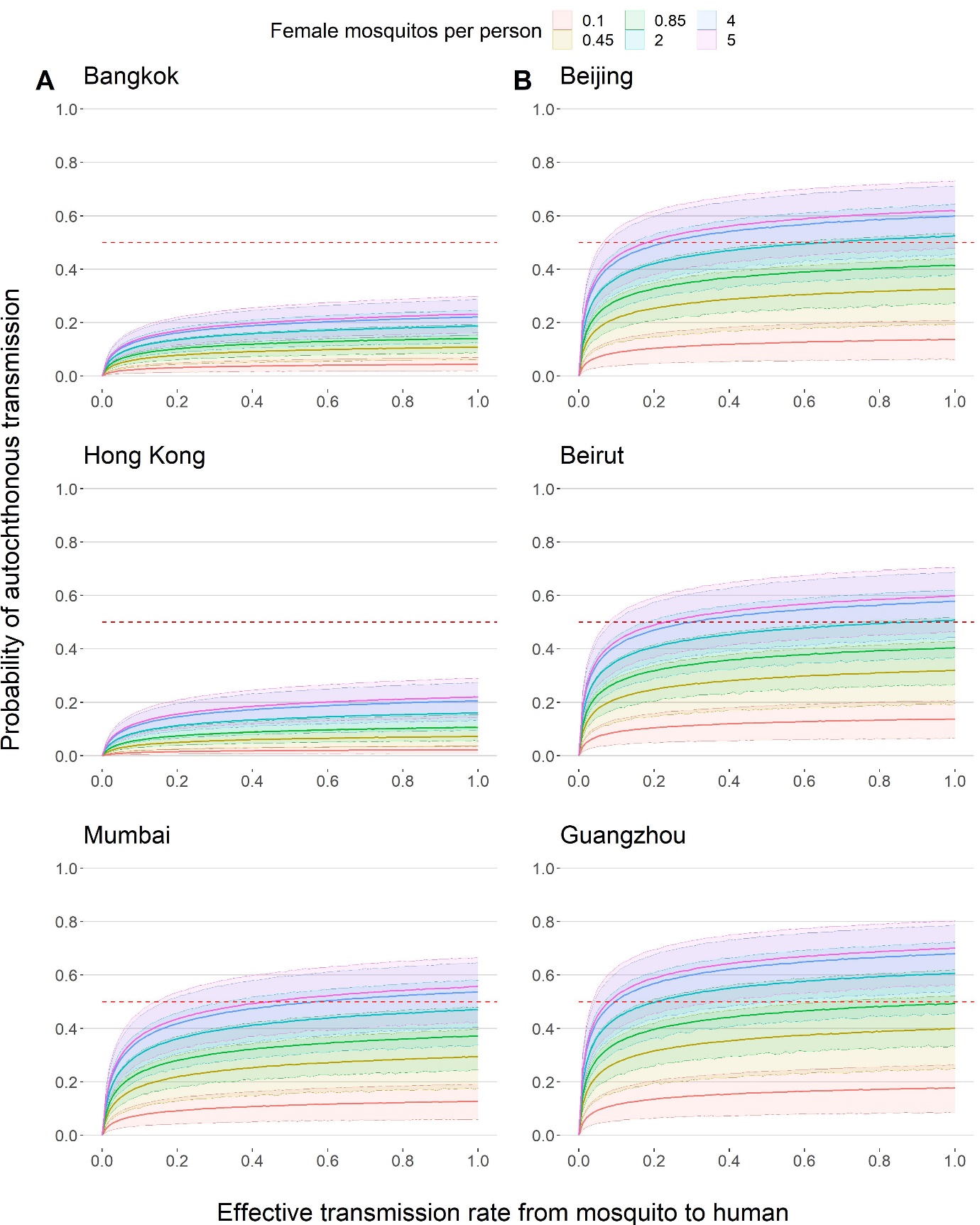


**Supplementary Figure 2:** Sensitivity analysis of the impact of increasing mosquito competency on the probability of autochthonous transmission at **A)** the average temperature in Bangkok, Hong Kong and Mumbai assuming transmission by Aedes aegypti and **B)** the maximum temperature in Beijing, Beirut and Guangzhou assuming transmission by Aedes albopictus. Also presented are probabilities at different values of the number of female mosquitos per human, where purple = 5, blue = 4, turquoise = 2, green = 0.85, yellow = 0.45 and red = 0.1. Thick coloured lines are the mean and shaded areas are the 95% confidence intervals. Dashed red horizontal line represents the point at which the probability of autochthonous transmission is below 0.5.

# **References for Supplementary Materials**

1. Dorigatti I, Hamlet A, Aguas R, et al. International risk of yellow fever spread from the ongoing outbreak in Brazil, December 2016 to May 2017. *Euro Surveill* 2017; **22**(28).

2. Johansson MA, Arana-Vizcarrondo N, Biggerstaff BJ, Gallagher N, Marano N, Staples JE. Assessing the risk of international spread of yellow fever virus: a mathematical analysis of an urban outbreak in Asuncion, 2008. *Am J Trop Med Hyg* 2012; **86**(2): 349-58.

3. Gaythorpe KA, Hamlet A, Cibrelus L, Garske T, Ferguson NM. The effect of climate change on yellow fever disease burden in Africa. *Elife* 2020; **9**.

4. Mordecai EA, Cohen JM, Evans MV, et al. Detecting the impact of temperature on transmission of Zika, dengue, and chikungunya using mechanistic models. *PLoS Negl Trop Dis* 2017; **11**(4): e0005568.

5. Luo XS, Imai N, Dorigatti I. Quantifying the risk of Zika virus spread in Asia during the 2015-16 epidemic in Latin America and the Caribbean: A modeling study. *Travel Med Infect Dis* 2020; **33**: 101562.

6. Aziz AT, Dieng H, Ahmad AH, et al. Household survey of container-breeding mosquitoes and climatic factors influencing the prevalence of Aedes aegypti (Diptera: Culicidae) in Makkah City, Saudi Arabia. *Asian Pac J Trop Biomed* 2012; **2**(11): 849-57.

7. Furuya H. Estimation of reproduction number and probable vector density of the first autochthonous dengue outbreak in Japan in the last 70 years. *Environ Health Prev Med* 2015; **20**(6): 466-71.

8. Almeida AP, Baptista SS, Sousa CA, et al. Bioecology and vectorial capacity of Aedes albopictus (Diptera: Culicidae) in Macao, China, in relation to dengue virus transmission. *J Med Entomol* 2005; **42**(3): 419-28.

9. Singh G, Tilak R, Kaushik SK. Bio-eco-social determinants of Aedes breeding in field practice area of a medical college in Pune, Maharashtra. *Indian J Public Health* 2019; **63**(4): 324-9.

10. Focks DA, Brenner RJ, Hayes J, Daniels E. Transmission thresholds for dengue in terms of Aedes aegypti pupae per person with discussion of their utility in source reduction efforts. *Am J Trop Med Hyg* 2000; **62**(1): 11-8.

11. Ahmad Zaki Z, Che Dom N, Ahmed Alhothily I. Efficacy of Bacillus thuringiensis Treatment on Aedes Population Using Different Applications at High-Rise Buildings. *Trop Med Infect Dis* 2020; **5**(2).

12. Wang JN, Hou J, Zhong JY, et al. Relationships between traditional larval indices and meteorological factors with the adult density of Aedes albopictus captured by BG-mosquito trap. *PLoS One* 2020; **15**(6): e0234555.

13. Liu H, Liu L, Cheng P, et al. Bionomics and insecticide resistance of Aedes albopictus in Shandong, a high latitude and high-risk dengue transmission area in China. *Parasit Vectors* 2020; **13**(1): 11.

14. Al-Abri SS, Kurup PJ, Al Manji A, et al. Control of the 2018-2019 dengue fever outbreak in Oman: A country previously without local transmission. *Int J Infect Dis* 2020; **90**: 97-103.

15. Lataillade LG, Vazeille M, Obadia T, et al. Risk of yellow fever virus transmission in the Asia-Pacific region. *Nat Commun* 2020; **11**(1): 5801.

16. Lloyd-Smith JO, Schreiber SJ, Kopp PE, Getz WM. Superspreading and the effect of individual variation on disease emergence. *Nature* 2005; **438**(7066): 355-9.

17. Ministério da Hotelaria e Turismo. Anuário de Estatística do Turismo de 2014. Tourism Statistics Yearbook 2014. 2014.

18. The World Tourism Organization. Benin: Country-Specific: Basic Indicators (Compendium) 2014 - 2018. 2019.

19. Ministere De La Culture Des Arts et Du Tourisme. Rapport Statistique De La 26e Edition Du FESPACO. Statistical Report of the 26th Edition of FESPACO. 2019.

20. The World Bank. Strategies for Urbanization and Economic Competitiveness in Burundi. Washington, DC. , 2015.

21. Institut National de la Statistique. Annuaire Statistique du Cameroun, Edition 2017. Cameroon Statistical Yearbook, 2017 Edition. 2017.

22. Ministère Du Tourisme Et De L'environnement. Statistique Du Tourisme Au Congo 2012 Annuaire. Congo Tourism Statistics 2012 Directory. . 2012.

23. World Tourism Organization. Cote D´ivoire: Country-Specific: Basic Indicators (Compendium) 2014 - 2018. 2019.

24. The World Bank. Ethiopia. In Makeda’s Footsteps: Towards a Strategy for Pro-Poor Tourism Development. Washington, DC, 2016.

25. Ghana Statistical Service. Trends in the Tourism Market in Ghana 2005 - 2014. 2017.

26. Instituto Nacional de Estatística e Censos. Guiné-Bissau em Números 2005. Guinea-Bissau in Figures 2005. 2005.

27. The World Bank. Kenya’s Tourism: Polishing the Jewel. Washington, DC, 2010.

28. World Tourism Organization. Niger: Country-Specific: Basic Indicators (Compendium) 2014 - 2018. 2020.

29. World Tourism Organisation. Nigeria: Country-Specific: Basic indicators (Compendium) 2014 - 2018. 2019.

30. The World Bank GoR. Future Drivers of Growth in Rwanda: Innovation, Integration, Agglomeration, and Competition. Washington, DC, 2020.

31. Ministere Du Toursime. Revue Annuelle Conjointe Du Tourisme. Joint Annual Review of Tourism. 2018.

32. World Tourism Organisation. Sierra Leone: Country-Specific: Basic Indicators (Compendium) 2014 - 2018. 2019.

33. Ministry of Natural Resources and Tourism. The 2017 International Visitors’ Exit Survey Report. 2017.

34. The World Bank. Togo Future Sources of Growth. Washington, DC, 2019.

35. The World Bank. Economic and Statistical Analysis of Tourism in Uganda. Washington, DC, 2013.

36. World Tourism Organisation. Zambia: Country-Specific: Basic Indicators (Compendium) 2014 - 2018. 2019.

37. World Tourism Organisation. Argentina: Country-Specific: Basic Indicators (Compendium) 2014 - 2018. 2019.

38. World Tourism Organisation. Bolivia: Country-Specific: Basic Indicators (Compendium) 2014 - 2018. 2019.

39. Ministério do Turismo. Estudo da Demanda Turística Internacional 2016. International Tourism Demand Study 2016. 2016.

40. Departamento Administrativo Nacional de Estadística. Encuesta de Viajeros Internacionales. International Traveller Survey. 2013.

41. World Tourism Organization. Ecuador: Country-Specific: Basic Indicators (Compendium) 2014 - 2018. 2020.

42. World Tourism Organization. French Guiana: Country-Specific: Basic Indicators (Compendium) 2014 - 2018. 2020.

43. World Tourism Organization. Guyana: Country-Specific: Basic Indicators (Compendium) 2014 - 2018. 2020.

44. World Tourism Organization. Panama: Country-Specific: Basic Indicators (Compendium) 2014 - 2018. 2019.

45. World Tourism Organization. Paraguay: Country-Specific: Basic Indicators (Compendium) 2014 - 2018. 2019.

46. Instituto Nacional De Estatística e Informática. Arribos y Pernoctaciones de Extranjeros en Establecimientos de Hospedaje Colectivo, Según Residencia Habitual de los Huéspedes. Arrivals and Overnight Stays of Foreigners in Collective Accommodation Establishments, According to the Habitual Residence of the Guests. 2018.

47. Algemeen Bureau voor de Statistiek. Milieustatistieken. Environmental Statistics. 2016.

48. World Tourism Organization. Trinidad And Tobago: Country-Specific: Basic Indicators (Compendium) 2014 - 2018. 2020.

49. World Tourism Organization. Venezuela: Country-Specific: Basic Indicators (Compendium) 2013 - 2017. 2019.

50. Kraemer MU, Sinka ME, Duda KA, et al. The global distribution of the arbovirus vectors Aedes aegypti and Ae. albopictus. *Elife* 2015; **4**: e08347.

51. Cattarino L, Rodriguez-Barraquer I, Imai N, Cummings DAT, Ferguson NM. Mapping global variation in dengue transmission intensity. *Science Translational Medicine* 2020; **12**(528): 1-11.

52. World Health Organisation Chikungunya in India, 2016.

53. World Health Organisation. Zika virus infection – India, 2017.

54. Pulmanausahakul R, Roytrakul S, Auewarakul P, Smith DR. Chikungunya in Southeast Asia: understanding the emergence and finding solutions. *Int J Infect Dis* 2011; **15**(10): e671-6.

55. Duong V, Dussart P, Buchy P. Zika virus in Asia. *Int J Infect Dis* 2017; **54**: 121-8.

56. Yue Y, Liu X, Xu M, Ren D, Liu Q. Epidemiological dynamics of dengue fever in mainland China, 2014-2018. *Int J Infect Dis* 2019; **86**: 82-93.

57. Ducheyne E, Tran Minh NN, Haddad N, et al. Current and future distribution of Aedes aegypti and Aedes albopictus (Diptera: Culicidae) in WHO Eastern Mediterranean Region. *Int J Health Geogr* 2018; **17**(1): 4.

58. Youssef M, El Zein S, Kanj S. Dengue fever in Lebanon: First confirmed case since 1945 and review from the region. *J Infect Dev Ctries* 2018; **12**(4): 286-9.

59. Luo L, Jiang LY, Xiao XC, et al. The dengue preface to endemic in mainland China: the historical largest outbreak by Aedes albopictus in Guangzhou, 2014. *Infect Dis Poverty* 2017; **6**(1): 148.

60. Zhang Z, Jing Q, Chen Z, et al. The increasing menace of dengue in Guangzhou, 2001-2016: the most important epicenter in mainland China. *BMC Infect Dis* 2019; **19**: 1002.

61. Wu D, Wu J, Zhang Q, et al. Chikungunya outbreak in Guangdong Province, China, 2010. *Emerg Infect Dis* 2012; **18**(3): 493-5.

62. Zhang J, Jin X, Zhu Z, et al. Early detection of Zika virus infection among travellers from areas of ongoing transmission in China. *J Travel Med* 2016; **23**(5).

63. Ma SK, Wong WC, Leung CW, et al. Review of vector-borne diseases in Hong Kong. *Travel Med Infect Dis* 2011; **9**(3): 95-105.

64. Humphrey JM, Cleton NB, Reusken CB, Glesby MJ, Koopmans MP, Abu-Raddad LJ. Dengue in the Middle East and North Africa: A Systematic Review. *PLoS Negl Trop Dis* 2016; **10**(12): e0005194.

65. Hussain R, Alomar I, Memish ZA. Chikungunya virus: emergence of an arthritic arbovirus in Jeddah, Saudi Arabia. *East Mediterr Health J* 2013; **19**(5): 506-8.

66. Lee H, Kim JE, Lee S, Lee CH. Potential effects of climate change on dengue transmission dynamics in Korea. *PLoS One* 2018; **13**(6): e0199205.

67. Kobayashi D, Murota K, Fujita R, et al. Dengue Virus Infection in Aedes albopictus during the 2014 Autochthonous Dengue Outbreak in Tokyo Metropolis, Japan. *Am J Trop Med Hyg* 2018; **98**(5): 1460-8.
